# Building leadership and managerial capacity for maternal and newborn health services

**DOI:** 10.1186/s12913-022-08448-7

**Published:** 2022-09-07

**Authors:** Gail Tomblin Murphy, Godfrey Mtey, Angelo Nyamtema, John LeBlanc, Janet Rigby, Zabron Abel, Lilian Teddy Mselle

**Affiliations:** 1 Nova Scotia Health and Dalhousie University WHO/PAHO Collaborating Centre on Health Workforce Planning and Research, 90 Lovett Lake Ct., Suite 201, Halifax, NS B3S 0H6 Canada; 2 Tanzanian Training Centre for International Health, Ifakara, Tanzania; 3 St Francis University College of Health and Allied Sciences, Ifakara, Tanzania; 4 Faculty of Medicine, Dalhousie University and IWK Health Centre, Halifax, NS Canada; 5 School of Nursing, Muhimbili University for Health and Allied Sciences (MUHAS), Dar es Salaam, Tanzania

**Keywords:** MNCH, Leadership, Management, Capacity building, Tanzania

## Abstract

**Background:**

Strengthening leadership and management is important for building an effective and efficient health system. This paper presents the findings from a L&M capacity building initiative which was implemented as part of a larger study aimed at improving maternal and newborn outcomes within primary health facilities in the Morogoro, Tanzania.

**Methods:**

The initiative, involving 30 stakeholders from 20 primary health facilities, 4 council health management teams and the regional health management team in the Morogoro region, provided leadership and managerial training through two 5-day in-person workshops, onsite mentoring, and e-learning modules. The initiative was evaluated using a pre-post design. Quantitative instruments included the ‘Big Results Now’ star-rating assessments and a team-developed survey for health providers/managers. The ‘Big Results Now’ star-rating assessments, conducted in 2018 (19 facilities) and 2021 (20 facilities), measured overall facility leadership and management capability, with comparisons of star-ratings from the two time-points providing indication of improvement. The survey was used to measure 3 key leadership indicators - team climate, role clarity/conflict and job satisfaction. The survey was completed by 97 respondents at baseline and 100 at follow up. Paired t-tests were used to examine mean score differences for each indicator. Triangulated findings from focus groups with 99 health providers and health management team members provided support and context for quantitative findings.

**Results:**

Star-ratings increased in 15 (79%) of 19 facilities, with the number of facilities achieving the target of 3 plus stars increasing from 2 (10%) in 2018 to 10 (50%) in 2021, indicating improved organizational performance. From the survey, team climate, job satisfaction and role clarity improved across the facilities over the 3 project years. Focus group discussions related this improvement to the leadership and managerial capacity-building.

**Conclusion:**

Improved leadership and managerial capacity in the participating health facilities and enhanced communication between the health facility, council and regional health management teams created a more supportive workplace environment, leading to enhanced teamwork, job satisfaction, productivity, and improved services for mothers and newborns. Leadership and managerial training at all levels is important for ensuring efficient and effective health service provision.

## Introduction

Leadership and governance, a core building block of the World Health Organization’s Health System Strengthening Framework [1], has been highlighted as a key factor in ensuring the health system is able to function effectively [2–5]. Without effective leadership, the health workforce cannot operate as a strong, cohesive team, collaborating to meet the needs of patients, nor can the health system provide the policies, governance, and infrastructure for the delivery of services. Health care professionals (clinical and administrative) need strong leadership and managerial capacities to be at the forefront of the transformational process. Leadership, leadership style, and management, including human resources management, are important to the health workforce, in terms of their work environment, improved performance, team culture, role clarity, job satisfaction, retention and recruitment [6–11].

In Sub-Saharan Africa, there has been a movement toward improving the leadership and governance health systems strengthening building block through the development of several leadership programs. The programs vary in terms of the target group (Ministry managers, health facility managers, frontline health care workers) and design (fellowship program, degree/certificate program, in-service training program), but collectively represent an effort to improve leadership across the health system [12–17].

Management capacity is also important to consider when seeking to improve the effectiveness of health systems. In Tanzania, health facilities have been assessed on various domains related to organizational management as part of a quality improvement program [18].

Specific to maternal, newborn, and child health (MNCH), ineffective leadership and governance have been cited as contributing factors to the challenges in achieving lower maternal mortality rates [19, 20]. There is evidence that organization of resources and poor leadership contribute more to mothers dying in childbirth than does poverty, with the problem of insufficient implementation of maternal health interventions being attributed to three interlinking factors: leadership and management, resources, and end-user related factors [21]. Success of maternal and child health interventions in improving quality of obstetric and newborn care has been attributed to 1) strong leadership in reproductive health, 2) accountability (governance) of both health providers and key decision makers to women, children and families, and 3) the presence of enabling policies [22]. Strengthening leadership and managerial capacities and capabilities within the health system are critical to enhance its ability to provide essential MNCH services to improve health outcomes among these groups.

The Accessing Safe Deliveries in Tanzania (ASDIT), aimed at identifying the requirements of scaling up Comprehensive Emergency Obstetrical and Newborn Care (CEmONC) in the Morogoro region of Tanzania, was conducted from 2015 to 2021, as part of the Innovation for Maternal and Child Health in Africa (IMCHA) programme [23]. During the early phase of the ASDIT project, the research team observed a gap in leadership and managerial capacity at the primary health facility (dispensary and health centre), district council, and regional levels. To address this gap, a leadership and management capacity-building initiative was implemented as part of the ASDIT project from 2018-2021.

This paper presents findings from the evaluation of the leadership and managerial capacity building initiative, which was carried out in the Morogoro region.

## Methods

### Leadership and managerial capacity building programme

The leadership and managerial capacity-building programme was designed to be a blended model comprising in-person workshops, e-learning modules and mentoring; informed by the observational findings during initial implementation of the ASDIT project, a project-specific health care provider survey at primary health facilities, and the results of the 2018 BRN assessment conducted by the Quality Assurance Unit of the Ministry of Health. The leadership and management programme for the study was developed to incorporate standard components of leadership, management and the health system, as listed in Table 1. Modules were developed by the team using its collective expertise, peer-reviewed literature, and other publicly available leadership workshops.

**Table 1:** Leadership and management capacity-building modules

Leadership	Management	Health System
Leadership Theory and Styles Time Management Leading Organizational Change Communication Decision-Making and Strategic Management Conflict Management Motivation & Empowerment	Performance Management HR Management Materials Management Direct Health Financing Planned Prevention Management Ethics, Professionalism & Code of Conduct	Health System Strengthening Health Planning Integrated Needs-based HRH Planning

The initiative involved 30 health providers and managers from the five health centres, which participated in the CEmONC training as part of the ASDIT project, and the 15 satellite dispensaries located in four district councils (Gairo, Morogoro, Kilosa and Mvomero), the four Council Health Management Teams (CHMTs), and the Regional Health Management Team (RHMT).

The programme was implemented through the provision of two face-to-face capacity building workshops (each done over a 5-day period) at the Tanzania Training Centre for International Health in Ifakara, development of four eLearning modules, and onsite mentoring by the research team in the 20 participating health facilities. The face-to-face leadership and management capacity building workshops were delivered in 2018 and 2020. Each workshop included 29 participants from 20 health facilities, 8 from four CHMTs and one from the RHMT, with some attending both workshops. To enhance uptake and implementation of transformational leadership at the primary health facilities, the first training workshop involved tasking the health facility participants with identifying the major leadership and management gaps that affected productivity (provision of care and outcomes) and for CHMT & RHMT participants to develop plans with three to four achievable objectives and strategic interventions, requiring little or no additional funds, for implementation in the following year. The second workshop in December 2020 included a review of key leadership concepts, human resources planning and management, and health system governance. The leadership and managerial e-Learning modules were introduced during the second workshop.

Onsite mentorship and coaching at the facility level occurred at various times between the two face-to-face workshops. During this mentorship, the study team addressed the identified gaps including inadequacies in human resources for health (HRH) management, gaps in maintenance of facility infrastructure & supply chain, low utilization of health management information systems (HMIS) data, enhanced community involvement and accountability, as well as suboptimal quality of health services & customer care.

### Study settings and design

The evaluation used a pre-post design to determine changes in workforce factors impacted by leadership - team climate, role clarity/conflict and job satisfaction, and the facilities’ BRN star-ratings, which assess the capability of the health facility management team to make the necessary changes to improve the quality of care provided at the health facility. The evaluation was conducted within the 20 participating health facilities, as described in the previous section, with all data collection occurring within these sites.

### Assessment of leadership and managerial capacity

The evaluation comprised quantitative and qualitative data collection, triangulated to assess the ability of the initiative to improve health facility leadership in both the provision of CEmONC services and overall facility management. Data used to assess leadership and management capability and capacity were collected using several tools – the government-led Big Results Now (BRN) Star Rating assessment tool, a team-developed semi-structured survey, and focus group discussions.

#### BRN Star Rating assessment tool

As part of the final assessment, the study team used the BRN Star Rating assessment tool to assess the overall quality of services at the facilities and compared results with the findings in the 2018 assessment conducted by the Ministry of Health, Community Development, Gender, Elderly and Children (MoHCDGEC), which was used as the baseline measure given that it was completed in August 2018. Aimed at improving the quality of healthcare, the BRN Star Rating system measures the performance of various healthcare facilities, with more stars indicating better quality of services. The BRN assessment covers a range of topics, including strengthening leadership capacity and practice, implementing strategic plans, succession planning, clarity on tasks, roles and responsibilities, regular supervisory visits, and improved supply chain mechanisms [18]. The tool was developed by the Tanzania MoHCDGEC quality assurance unit through a consultative process [18].

#### Survey of health care providers and managers

Baseline survey data were collected in March & April 2018, prior to the development of the leadership training modules. The survey for health providers collected data on care providers’ perceptions on leadership and managerial competencies, focusing on the following domains: staff development or skills improvement opportunities; team climate of the units and facilities where the respondents worked; staff role clarity; and job satisfaction. The questions on team climate, role clarity and job satisfaction were measured using validated scales from previous work by one of the co-principal investigators [24]. No formal pilot was conducted. The survey of health care workers focused on workers on the maternal and newborn units at the 5 health care facilities and 15 dispensaries. Members of the research team travelled to each facility for one day and all health providers on these units who were working on the day of the survey administration were invited to participate. The survey was provided in Kaswahili and the team was there to clarify questions for the respondent. Participation was voluntary and they were informed they could choose not to answer any question or withdraw at any time without any negative consequence. Completion of the survey took approximately 20 minutes.

#### Focus groups

Qualitative data on leadership were gathered during the 24 focus groups conducted at one time-point (2019) as part of the primary ASDIT study. The focus group guide was developed by the research team, based on the objectives of the ASDIT program. Using purposive recruitment, potential participants for the focus groups were identified in two ways. The ASDIT principal investigator from the Tanzanian Training Centre for International Health informed the health management teams. The nurses in-charge at each health centre identified the health care providers who had received the CEmONC training and community members who had received services in the facilities for two or more years. Two or more years was chosen as the selection criteria as it was felt that these individuals would be able to reflect on the changes in the facilities over the project period. Once identified, potential participants were approached by the lead qualitative researcher and informed of the purpose of the study, issues of confidentiality and for the community members, the voluntary nature of their participation. Health providers who had received the training and health management teams were required to participate with the understanding that this was part of their involvement with the ASDIT project.

The focus groups were held with health care providers at each of the health centres receiving CEmONC training (5 groups, 29 people in total), the respective health facility management teams (HFMT) (5 groups, 40 people in total), women and men attending the five project health centres for maternal and child health services (5 groups each; total of 40 women and 30 men) and Council Health Management Teams (4 groups, 30 p in total). All discussions were conducted in Kiswahili. The lead qualitative researcher (a midwife) and an associate (a physician) facilitated each of the group discussions. Only the findings from the health providers and health management teams (14 groups) were included in the analysis of leadership. Qualitative data were also derived from informal discussions with stakeholders during mentoring visits and regularly scheduled meetings of the council and health facility management teams. All data were used to complementarily reflect on the success of the leadership and management capacity building process during this initiative and to generate key messages.

### Data analysis

#### BRN star-ratings

For each domain, the percentage of indicators which were achieved were scored and then the number of stars were calculated based on the lowest percentage score among the domains.

#### Surveys

Survey data were analysed using descriptive analysis in STATA. For each selected quantitative indicator, the difference between 2018 and 2021 was tested using paired t-tests. No variables were controlled. Due to the small sample size, facility specific and multi-level analyses were not possible.

#### Focus group discussions

Audio-recorded focus group discussions were transcribed verbatim and translated from Kiswahili to English. Translation accuracy was cross-checked by the Tanzanian qualitative component lead. Qualitative content analysis [25] was used to analyze the test along pre-determined themes (the objectives of the project). The analysis process was led by the qualitative lead and multiple coding was done to ensure rigour. Group consensus was reached through research team discussions of common and emerging categories.

## Results

### Characteristics of participants

BRN star-ratings: Nineteen of the 20 health facilities were assessed in 2018 and all 20 participating facilities were assessed in 2021. Health provider survey: All 20 health facilities participated in the baseline and final assessments. The health care provider survey was completed by 94 health care providers and managers at baseline (2018) and 100 at follow up (2021). Only 15.6% of respondents completed both surveys. Focus groups: Ninety-nine health care providers and management team members participated in the 2019 ASDIT focus groups.

The distribution of all respondents is presented in Table 2.

**Table 2:** Distribution of participants for each data collection tool

**BRN Star-rating**	**2018 assessment**	**2021 assessment**
Participating health facilities	*N* = 19	*N* = 20
Health centre	21%	25%
Dispensary	79%	75%
**Surveys of health providers & managers**	**2018 survey**	**2021 survey**
Descriptor	*N* = 94	*N* = 100
Profession/ role*		
Facility/unit manager	5%	21%
Registered nurse/Enrolled nurse	39%	37%
Midwife	16%	19%
Clinical officer/Medical attendant	31%	31%
Other (i.e., other medical professional, laboratory technician, assistive personnel, etc.)	22%	21%
Gender		
Female	69%	63%
Male	31%	37%
Age group		
20 – 29	34%	15%
30 – 39	23%	57%
40 – 49	21%	14%
50+	21%	15%
**Focus Groups**	**2019** *N* = 99	
Gender		
Female	54%	
Male	46%	
Age group		
24 – 34	45%	
35 – 45	28%	
46 – 56+	26%	
Role		
Trained health care provider	29%	
Facility manager	40%	
CHMT member	30%	

*Total % >100 due to people having multiple roles within (e.g., clinical and administrative)

### Leadership and management capability and capacity

#### BRN Star-ratings

**Fig. 1:**
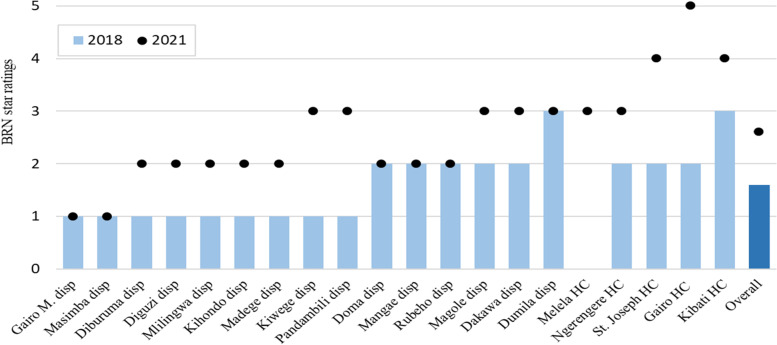
BRN star-ratings per participating sites, 2018 and 2021. Note: disp = dispensary, HC = health centre

The BRN star-ratings measured the overall facility leadership and management capability. The overall BRN Star-ratings increased in 15 (79%) of the 19 participating facilities (one facility did not have 2018 data provided to the team), with the number of facilities achieving the target of 3 plus star increasing from 2 (10%) in 2018 to 10 (50%) in 2021 (Fig. 1). Among these facilities, the overall mean of the star ratings increased from 1.6 (95% CI 1.3 – 2.0) in 2018 to 2.6 (95% CI 2.1 – 3.1) in 2021 (Fig. 1).

**Fig. 2:**
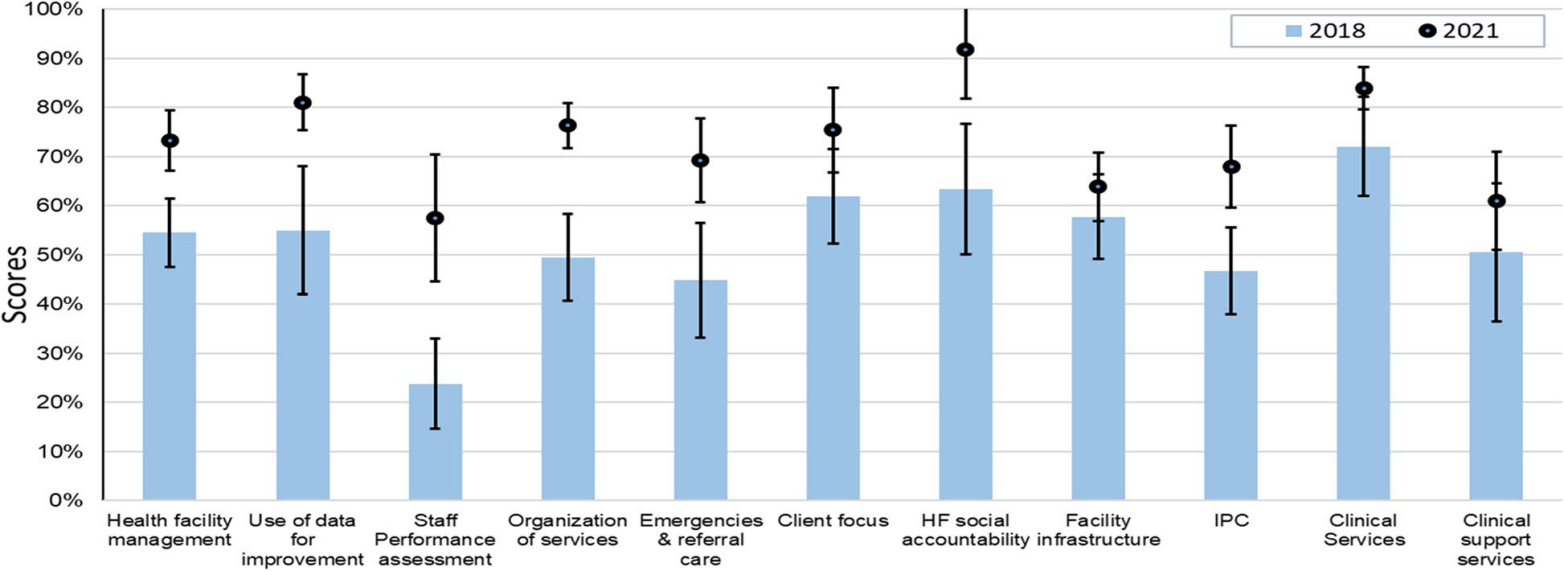
Scores of the BRN key areas before and after leadership and management capacity building programme

Seven out of 11 BRN indicators (key areas) improved significantly (Fig. 2). These included health facility management, use of facility data for planning and improvement, staff performance assessment, organization of services, handling of emergencies and referrals systems, social accountability at the health facility, and infection prevention and control.

While there was a substantive improvement during the project, for many facilities, barriers such as lack of financial resources and limited facility staffing inhibited their ability for even greater improvement.

#### Survey of health providers and managers

The survey measured three key leadership indicators of team climate, role clarity/conflict and job satisfaction, with improved team leadership, role clarity and job satisfaction indicating improved leadership capacity. In this section, results from the analysis of the focus groups are inserted to provide context and support for the survey findings.

##### Team climate

**Fig. 3:**
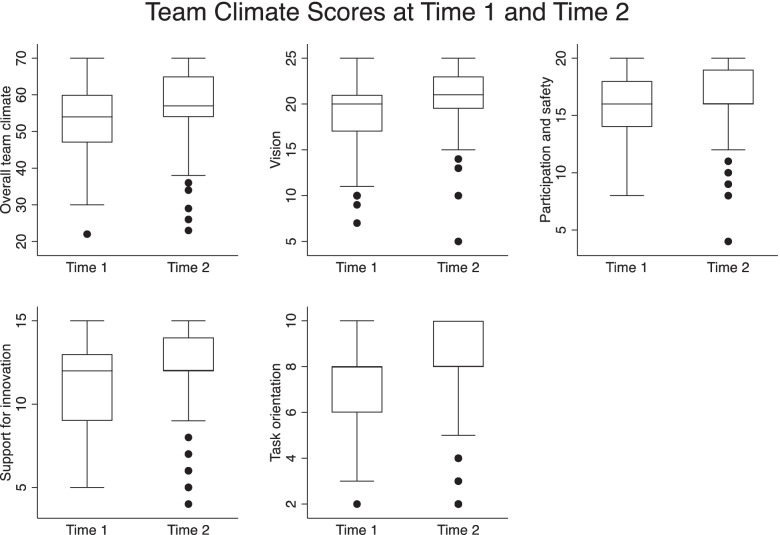
Team climate scores, 2018 and 2021

Team climate refers to the employee’s shared perception of organisational events, practices, and procedures. The Team Climate Inventory short version [26, 27] was used to measure overall team climate as well as the four sub-constructs – vision, participation & safety, support for innovation and task orientation. Overall team climate improved significantly (*p* = .005) between baseline (M=52.6) and end measurement (M=57.4), as did all sub-scales, vision (M=18.7; M=20.8 *p*=.000); participation (15.4,16.5; *p*=.021); support for innovation (10.8, 12.1; *p*=.002) and task orientation (7.4, 8.1; *p*=.021), indicating a positive effect of the leadership and managerial capacity building (Fig. 3).

Improved team climate was also reflected in the focus group discussions with health care providers. Participants reported that the CEmONC training helped them to acquire strong team spirit and they became highly committed to work collaboratively in providing emergency obstetric and newborn care.

The spirit of teamwork and commitment among staff have increased and continue to improve every day, unlike in the past (…). For example, in the health centre, providers are now confidently and collectively attending the emergenc[y] because they have skills and they are capable of managing such emergencies. This is an outcome of the training. (Health care provider)

Trained health care providers and CHMT members also reflected that their acquired capacity for enhanced teamwork and the improved morale reduced workload and improved client outcomes. It was reported by the health centre managers that health care providers who received the leadership training improved their leadership skills, attained leadership roles, and were better able to mentor their subordinates. In addition, participation and supervision skills of council members improved due to the leadership training.

(…) they are willing and ready to work as a team. Recently, I observed the staff gathered to attend an emergency as a team and thereafter, they transferred the woman to the hospital for further management. I believe it was because of training where they learnt the importance of working as a team. (CHMT member).

Leadership training has helped us in different ways. In the first place, in organizing our subordinate’s environment ready for learning new things (…). Our coordination skills have also improved, and we can provide constructive feedback that cannot [negatively] affect others in the work environment. We are thankful they are perceived well and things are going well (HFMT member).

##### Job satisfaction

**Fig. 4:**
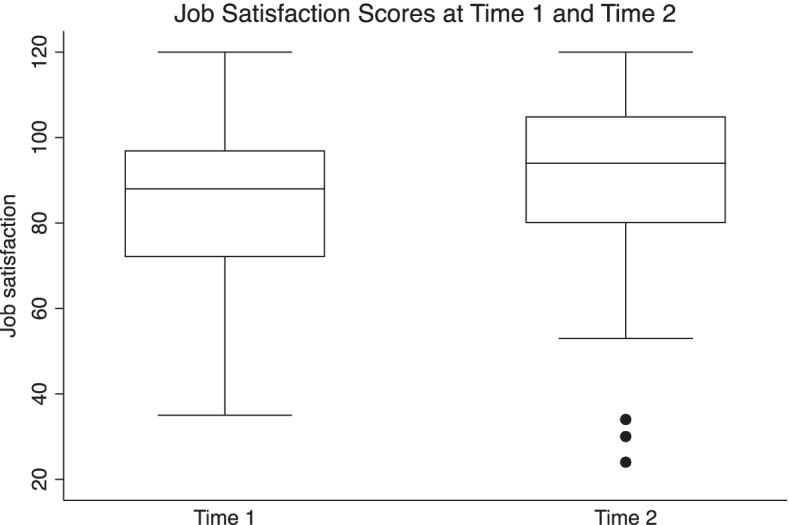
Job satisfaction scores, 2018 & 2021

Job satisfaction is one of the key goals of human resources management. Job satisfaction was measured through degree of agreement on fourteen statements relating to care coordination, communication, control, expectations, involvement in decision-making, time to do job, trust in facility, facility support, work safety, work-life balance, social opportunities, responsibilities, overall job, leadership and working in the profession. Although not significant, overall job satisfaction increased slightly, reflecting movement toward a more positive work environment for the health care providers in the participating sites (Fig. 4).

##### Role clarity

**Fig. 5:**
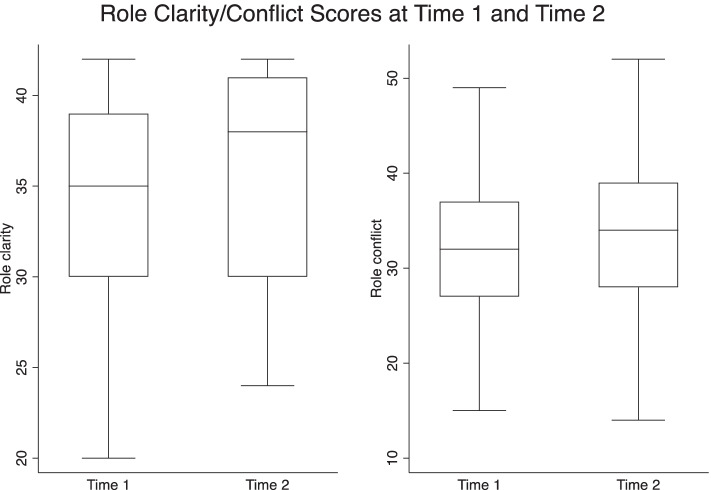
Role clarity scores, 2018 and 2021

Role clarity refers to how an individual perceives their role within the organization (tasks, responsibilities) and role conflict is one’s perception of their role as being in conflict with the expectations. Over the two years, role clarity significantly increased among provider at the participating sites (Fig. 5). Paradoxically, there was also a slight increase in role conflict, although not significant. The reason for this is not clear.

The role of the management in the team and improving supervision were additional themes identified by participants in the focus groups.

When there is an emergency, the administration team must be given information and they help to organize the team and communicate with staff to help. Therefore, the leader gets involved in that way. The leaders organise the roster and inform staff. (Health facility manager)

After the leadership training, we note a lot of changes. Great improvement has been made in terms of participation in the CHMT and supervision of community issues within the health centres. (CHMT member).

Overall, the focus group findings show that perception of the leadership and management training and its effect on the health care providers’ ability to provide better services was positive. In particular, the perceived improvement in teamwork and providing mentorship supports the finding of improved team climate as per the health provider survey.

(…) one staff who participated in the leadership training came back and he was given a leadership role. He managed it very well. (…) You look at him like you really think this is a good leader and he can really lead. Even if you are his immediate leader, he has confidence to ask you to do this and that. They have also been our mentors. They have helped to shape even other staff as they lead by example (…). (HFMT member).

## Discussion

The aim of this capacity-building initiative was to address the observed gaps in leadership and management within the participating centres, which occurs at every level of the health system and provides the structure within which the health workforce provides quality health care. The results of this evaluation are discussed in terms of how this initiative addressed areas of leadership and management capacity overall, health workforce factors, and quality of health care provision while also providing insight into continued areas for improvement and scaling up beyond the initial participant group. The limitations of the evaluation are also discussed.

### Improving capacity at all levels

In Tanzania, the health system has been decentralized so that the regions and districts have more autonomy. In addition, all health facilities are responsible for their own budgeting, although any changes in their budget allocations need to be approved at the higher levels. Findings from the baseline survey indicate that majority of the key managers, i.e., heads of primary health facility and members of the council health management teams, are given leadership and managerial responsibilities without prior training. These findings reflect the situation in Tanzania and as such, provide justification and a model for scaling up the initiative countrywide. The ability to build leadership and management capacity across the levels of management (RHMT, CHMT & HFMT) in the Morogoro region has created an enhanced environment for shared decision-making, accountability, and improved the ability for improving the star-rating (i.e., performance) among the participating centres. Our findings are supported by a study on the BRN star-ratings by Gage et al [28], which found one of the key influences of star-rating improvements was district administration. The involvement of the district quality improvement coordinators for the national star-rating program in the capacity-building initiative is an important link for sustainability of the initiative at the district level. This involvement is one way that scaling up nationally can be accomplished and sustained.

### Leadership and the health workforce

Important to leadership and management for the health workforce is the ability to manage change and cultivate an environment for achieving the behaviour change required. Strengthening leadership requires intervention at individual, team and system levels [4, 29] to have a sustainable impact on the ability for the health system to provide safe and quality health services. In addition, distributed and collective leadership within health care organizations have been shown to contribute to a greater alignment between clinicians and managers [28] and can impact the ability to influence change [10]. A blended approach is thought to be beneficial to ensuring leadership, management and governance training needs for health professionals are met [12, 30]. The results and experiences of this project support these previous findings that a blended approach, in this case comprising face-to-face, e-learning modules, and on-site mentoring, is useful for ongoing development of skills within the workplace which in turn, will continue to have a positive effect on the functioning of the health facilities.

During the workshops, transformational leadership was highlighted as the most effective leadership approach. It has been demonstrated that leaders utilizing this approach are able to motivate their colleagues, improve teamwork, and increase job satisfaction more effectively [8, 9, 31, 32]. Consistent with these studies, the increased team climate, role clarity, and job satisfaction found in this study, while not significant, illustrates a positive outcome of the initiative as well as the need to have ongoing capacity development to ensure sustained effect.

The importance of not isolating leadership capacity strengthening from other improvement activities has been highlighted by Pfeiffer et al, [12] where front-line workers in Ghana were supported to develop and implement leadership projects within the context of their workplaces. Negero et al [33] in a systematic literature review on HRH interventions, found that four or more health workforce initiatives were more likely to see improved quality of maternal and newborn care and that local financial and administrative policy and planning to improve operational activities were more effective. In this project, the inclusion of the leadership and managerial capacity-building within the context of improving the capacity to provide comprehensive (in health centres) and basic (in dispensaries) emergency obstetrical and neonatal care services was important to the overall aim of improving the quality of maternal and newborn health care in these facilities.

### Improved quality of MNCH services

These findings suggest that capacity building in leadership and management for the RHMT, CHMT and HFMT levels contributed to the capability of the primary health facilities to provide CEmONC services, such as an improved supply chain and more efficient budgeting. With the inclusion of the Star rating indicators as part of the leadership and management capacity building, there is a solid integration of local, regional and national efforts to enhance planning and to improve the quality of health services at each of the facilities. Additionally, it is important to link this with health workforce planning processes to ensure properly integrated service and workforce planning occurs at the national, regional and district levels.

### Areas for continued improvement

The project identified areas for continued improvement related to leadership and managerial capacity within the facilities and the CHMTs, including the need to improve the skills-set of leaders (both clinical and administrative), improving the implementation and monitoring of policies and activities, and improving the management of financial administration, safety measures, chain-supply management, staffing, and infrastructure maintenance/ improvement.

Leadership across the system (including clinical and administrative sectors) is required for evidence-informed decision- and policy making, particularly with the decentralization of financial and material resources as well as key services to the primary health facility level. Furthermore, implementation of leadership and management at the health facility level may be affected by poor leadership styles at higher levels. For example, during this project, consultations with management teams highlighted that currently, a facility manager/chair must travel (often many kilometres) to get approvals for changes or additional budgetary expenditures. If there is an issue with the request, then the person is sent back to get clarifications and often, the person may not return to get the approval. This practice is seen as a barrier to the facilities’ ability to utilize their finances appropriately. A solution is to have a cascaded approval process for expenditure authorizations so that the facility can appropriately utilize its finances to ensure there is adequate infrastructure and staffing in place to effectively provide services. Effective communication and leadership across the levels would allow for this.

The areas for improvement in leadership and management need to be addressed through additional capacity building, with a national mandate for capacity-building programs to occur at the regional, district, council, and facility levels. The BRN Star Rating System has been implemented by the government to ensure all health care facilities can monitor their facility’s capacity using a set of core indicators. Leadership and management capacity building can be aligned with this mechanism as a way of measuring progress, although the BRN assessment may need to incorporate additional indicators directly measuring leadership. The BRN star rating strategy has been effective in identifying areas for improvement and should be strengthened in terms of frequency and length of visits as well as face-to-face time with facilities staff to review core indicators, evaluate changes since the last visit, and plan future changes.

Through the implementation of leadership and management capacity building workshops and continuous mentorship activities, there was a positive effect on implementation of the BRN star rating improvement plans for each health facility. In particular, the activities strengthened the managerial and supervisory relationships among the health management teams. Continuous mentoring, provision of e-learning opportunities, and refresher workshops are important for the continued development of leadership and managerial capacity in not only these health facilities, but in all the health facilities in the Morogoro region. In addition, all health facilities in the region would benefit from sustained leadership development at the CHMT and the RHMT levels.

### Scaling up leadership and managerial capacity building initiatives

As a result of the leadership and managerial capacity strengthening workshops and continuous mentorship activities, many changes were made in the participating facilities, leading to an improvement in the key domains and overall BRN star ratings. These changes within the facilities demonstrate the potential of transformational leadership in changing health systems in resource limited countries, as found in other studies [16, 34, 35]. It is important to note that most of the health facility managers in this project did not receive any training/orientation on leadership prior to being given their leadership role, which may also be the case in other districts/regions of the country. As such, the full impact of transformational leadership on strengthening the Tanzanian health system will only be achieved through scale up of leadership capacity programs across its facilities.

While much of the emphasis was on building leadership capacity, the project included management capacity as there was an observed need for improvement in this area as well. In a scoping review of leadership programs in Sub-Saharan Africa, Johnson and colleagues [36] found that many of the programs combined leadership and management capacity building, which they concluded was reasonable, given than the leaders within the health facilities are also, in many instances, managers who must oversee both change and performance.

Based on the findings of this study, actions that intersect both the health system building blocks and levels of the system are needed to enhance excellence and improve health system performance. This will be aided by ensuring leadership and management capacity through ongoing access to capacity building programs, mentoring to sustain leadership for change, and effective communication and accountability across the levels of the system. In Tanzania, effective leadership through improved communication and interactions between the HFMTs, CHMTs and RHMTs, as well as between RHMTs and the Ministry, are critical to transformative change, as well as the systematic and robust use of the health information system to drive the service and policy decisions. The lack of effective leadership and management is a barrier to health system improvement; building capacity in leadership and management becomes an enabler to health system improvement.

In stakeholder meetings related to the IMCHA program, the MoHCDGEC and the President’s Office Regional Administration and Local Government (PORALG) acknowledged existence of huge gaps in leadership and management and expressed interest to support interventions that focus on strengthening these skills at the primary health facilities and CHMTs. This interest is also based on the decentralization of most of the health care services that do not need specialized attention to primary health care facilities. In doing so human and material resources are also decentralized. Given the interest expressed by the MoHCDGEC and PORALG, efforts are also under way to review and customize the project eLearning modules for scale up in Tanzania. After review together with other stakeholders, the e-modules will be uploaded on the government eLearning platform for scale up in the country.

### Limitations

The findings from this study have several limitations. As the number of facility staff completing surveys at both baseline and follow up was low, a longitudinal analysis was not possible. This limits the ability to determine if changes occurred as a sole result of the initiative. Indicators such as team climate could be affected by new people on staff who have different attitudes, training, and behaviours. However, the qualitative findings around team climate support the survey findings, thus mitigating this potential limitation. Secondly, the surveys had to be translated into Swahili and as such it is hard to determine the impact language and context had on the responses. This may, in part, explain the dichotomous findings of improved role clarity and at the same time, increases in role conflict. Finally, the study did not have time to fully assess the role of the e-learning modules in assisting with the development of leadership and managerial capacity. Ongoing monitoring and evaluation of the regional mentorship for leadership and managerial capacity and the effectiveness of the developed e-learning modules should be conducted. Since the e-learning modules will be available from a national database, there is the potential for extended assessment.

## Conclusion

This project addressed the link between leadership and managerial skills and provision of emergency obstetric and neonatal care services. Improving leadership and managerial capacity in primary health facilities creates a more supportive workplace environment, leading to enhanced productivity and improved MNCH services.

Proposed actions that can be taken

1. Align leadership and management capacity building with the BRN star-rating system to ensure that all facilities have access to this capacity building.
2. Scale up capacity building leadership and managerial programs to management and provider staff within facilities.
3. Clarify tasks, roles, and responsibilities among administration of health facilities and health management teams.
4. Evaluate the effectiveness of the e-learning modules developed in this study.

## Data Availability

The survey data used and analysed during the current study are available from the corresponding author on reasonable request. The BRN star-rating assessment data used during the current study were provided by the United Republic of Tanzania Ministry of Health, Community Development, Gender, Elderly and Children and may not be made public. Requests for this data may be made to the Ministry.

## Abbreviations

ASDIT: Accessing Safe Deliveries in Tanzania
BRN: Big Results Now
CEmONC: Comprehensive emergency obstetric and newborn care
CHMT: Council health management team
HFMT: Health facility management team
HMIS: Health management information system
HRH: Human resources for health
MoHCDGEC: Ministry of Health, Community Development, Gender, Elderly and Children
MNCH: Maternal, newborn and child health
PORALG: President’s Office Regional Administration and Local Government
RHMT: Regional health management team
